# GlucoGenes®, a database of genes and proteins associated with glucose metabolism disorders, its description and applications in bioinformatics research

**DOI:** 10.18699/vjgb-24-107

**Published:** 2024-12

**Authors:** V.V. Klimontov, K.S. Shishin, R.A. Ivanov, M.P. Ponomarenko, K.A. Zolotareva, S.A. Lashin

**Affiliations:** Research Institute of Clinical and Experimental Lymphology – Branch of the Institute of Cytology and Genetics of the Siberian Branch of the Russian Academy of Sciences, Novosibirsk, Russia; Research Institute of Clinical and Experimental Lymphology – Branch of the Institute of Cytology and Genetics of the Siberian Branch of the Russian Academy of Sciences, Novosibirsk, Russia; Institute of Cytology and Genetics of the Siberian Branch of the Russian Academy of Sciences, Novosibirsk, Russia; Institute of Cytology and Genetics of the Siberian Branch of the Russian Academy of Sciences, Novosibirsk, Russia; Institute of Cytology and Genetics of the Siberian Branch of the Russian Academy of Sciences, Novosibirsk, Russia; Research Institute of Clinical and Experimental Lymphology – Branch of the Institute of Cytology and Genetics of the Siberian Branch of the Russian Academy of Sciences, Novosibirsk, Russia Institute of Cytology and Genetics of the Siberian Branch of the Russian Academy of Sciences, Novosibirsk, Russia

**Keywords:** gene, protein, diabetes mellitus, hyperglycemia, hypoglycemia, glucose variability, database, phylostratigraphic index;, single nucleotide polymorphism, ген, белок, cахарный диабет, гипергликемия, гипогликемия, вариабельность глюкозы, база данных, филостратиграфический индекс, однонуклеотидный полиморфизм

## Abstract

Data on the genetics and molecular biology of diabetes are accumulating rapidly. This poses the challenge of creating research tools for a rapid search for, structuring and analysis of information in this field. We have developed a web resource, GlucoGenes®, which includes a database and an Internet portal of genes and proteins associated with high glucose (hyperglycemia), low glucose (hypoglycemia), and both metabolic disorders. The data were collected using text mining of the publications indexed in PubMed and PubMed Central and analysis of gene networks associated with hyperglycemia, hypoglycemia and glucose variability performed with ANDSystems, a bioinformatics tool. GlucoGenes® is freely available at: https://glucogenes.sysbio.ru/genes/main. GlucoGenes® enables users to access and download information about genes and proteins associated with the risk of hyperglycemia and hypoglycemia, molecular regulators with hyperglycemic and antihyperglycemic activity, genes up-regulated by high glucose and/or low glucose, genes down-regulated by high glucose and/or low glucose, and molecules otherwise associated with the glucose metabolism disorders. With GlucoGenes®, an evolutionary analysis of genes associated with glucose metabolism disorders was performed. The results of the analysis revealed a significant increase (up to 40 %) in the proportion of genes with phylostratigraphic age index (PAI) values corresponding to the time of origin of multicellular organisms. Analysis of sequence conservation using the divergence index (DI) showed that most of the corresponding genes are highly conserved (DI < 0.6) or conservative (DI < 1). When analyzing single nucleotide polymorphism (SNP) in the proximal regions of promoters affecting the affinity of the TATA-binding protein, 181 SNP markers were found in the GlucoGenes® database, which can reduce (45 SNP markers) or increase (136 SNP markers) the expression of 52 genes. We believe that this resource will be a useful tool for further research in the field of molecular biology of diabetes.

## Abstract (ru)

Данные в области генетики и молекулярной биологии сахарного диабета стремительно накапливаются. Это ставит задачу создания исследовательских инструментов для быстрого поиска, структурирования и анализа информации в этой области. Мы разработали базу данных о генах и белках человека, ассоциированных с высоким уровнем глюкозы (гипергликемией), низким уровнем глюкозы (гипогликемией) и обоими нарушениями. Сведения были собраны с помощью текст-майнинга научных публикаций, проиндексированных в PubMed и PubMed Central, и анализа генных сетей гипергликемии, гипогликемии и вариабельности гликемии, выполненного с помощью биоинформатической системы ANDSystems. Созданный ресурс (GlucoGenes®) доступен по адресу: https://glucogenes.sysbio.ru/genes/main. Ресурс предоставляет информацию о генах и белках, связанных с риском развития гипергликемии и гипогликемии; регуляторных молекулах с гипергликемической и антигипергликемической активностью; генах, экспрессия которых повышается при высоком и/или низком уровне глюкозы; генах, экспрессия которых снижается при высоком и/ или низком уровне глюкозы, а также о молекулах, связанных с нарушениями метаболизма глюкозы иным образом. На основе ресурса проведен эволюционный анализ генов, ассоциированных с нарушениями метаболизма глюкозы. Результаты анализа выявили значительное увеличение (до 40 %) доли генов, имеющих филостратиграфический индекс (phylostratigraphy age index, PAI), соответствующий времени происхождения многоклеточных организмов. Анализ консервативности последовательностей белков по индексу дивергенции (divergency index, DI) показал, что большинство соответствующих генов высококонсервативны (DI < 0.6) или консервативны (DI < 1). При анализе однонуклеотидного полиморфизма (SNP) в проксимальных районах промоторов, влияющих на сродство ТАТА-связывающего белка, в базе данных GlucoGenes® найден 181 SNPмаркер, который может снижать (45 SNP-маркеров) или повышать (136 SNР-маркеров) экспрессию 52 генов. Мы полагаем, что разработанный ресурс станет полезным инструментом для дальнейших исследований в области молекулярной биологии диабета.

## Introduction

Diabetes is one of the most common and socially significant human diseases. According to experts from the International Diabetes Federation, the number of people living with diabetes worldwide reached 537 million in 2021 and is expected to rise to 783 million by 2045. In addition, more than 540 million people have impaired glucose tolerance (International Diabetes Federation, 2021).

In recent years, significant progress has been made in understanding the molecular mechanisms underlying the development of diabetes and its complications. Genome-wide association studies have identified a number of novel genetic loci that modulate the risk of diabetes and diabetic complications in European and Asian populations. Proteomics, metabolomics and multiomics studies have shed light on the molecular basis of disease pathogenesis (Langenberg, Lotta, 2018; Lyssenko, Vaag, 2023; Shojima et al., 2023).

At the same time, the effects of exposure to high glucose concentrations on the regulation of gene expression in different tissues have been identified (Vaulont et al., 2000; Hall et al., 2018; Vega et al., 2020; Zhang S. et al., 2021). It has been shown that the effects of high glucose levels on gene expression can be prolonged and exacerbated by epigenetic modifications. This mechanism is considered to be important for the phenomenon of metabolic memory and the development of diabetic complications (Dhawan et al., 2022). Abnormally low glucose levels are also associated with a number of biochemical shifts. These shifts are primarily related to the response of the cardiovascular and nervous systems to hypoglycemia (Hanefeld et al., 2016; Rehni, Dave, 2018).

The molecular effects of repeated episodes of high and low glucose levels, which characterize the phenomenon of high glycemic variability (GV), attract increasing attention. Elevated GV has been found to increase the risk of microvascular and macrovascular diabetic complications and is associated with increased all-cause and cardiovascular mortality (Ceriello et al., 2019; Wilmot et al., 2019). At the molecular level, the pathophysiological changes associated with high GV are realized through increased or decreased expression of a large number of genes and altered activity of signaling pathways such as PI3K/Akt, NF-κB, MAPK (ERK), JNK and TGF-β/Smad (Klimontov et al., 2021b).

Given the vast number and diversity of molecular changes in diabetes, advanced analytical tools are necessary to form a comprehensive and holistic understanding of the disease’s pathogenesis. Artificial intelligence, bioinformatics, and integrative systems biology provide new opportunities for studying complex diseases such as diabetes (Nielsen, 2017; Klimontov et al., 2021a; Orlov et al., 2021; Putra et al., 2024).

A promising approach in this field is the analysis of gene networks, i. e. groups of genes that function in a coordinated manner, interact with each other, and determine specific phenotypic traits of an organism (Kolchanov et al., 2013). Previously, using text mining of scientific publications indexed in the PubMed and PubMed Central (PMC) databases, along with bioinformatic analysis, we reconstructed gene networks associated with glucose metabolism disorders (GMDs): hyperglycemia, hypoglycemia, and GV, gathering a large amount of data on molecules and proteins related to these metabolic disorders in some way (Saik, Klimontov, 2020–2022).

Based on the obtained data, a database was created containing information about genes and proteins associated with GMDs: hyperglycemia, hypoglycemia, and both conditions. In this article, we present a description of the database’s capabilities and provide the results of two bioinformatics studies conducted using it. The first study involved an evolutionary analysis of GMD genes, and the second involved an analysis of single nucleotide polymorphisms (SNPs) in 90 base-pair proximal regions of human gene promoters associated with GMDs.

## Materials and methods

Development of the web resource. The material for creating the GlucoGenes® web resource was previously accumulated during the reconstruction and analysis of gene networks for hyperglycemia, hypoglycemia, and GV, conducted using ANDSystem, a bioinformatics tool (developed at Institute of Cytology and Genetics SB RAS; access: https://anddigest. sysbio.ru/index.php). ANDSystem constructs associative gene networks based on text mining of scientific publications indexed in the PubMed and PMC databases (Ivanisenko et al., 2015, 2019). The details of the analysis of GMD gene networks generated using ANDSystem were described previously (Saik, Klimontov, 2020–2022).

A relational data model and the PostgreSQL database management system (https://www.postgresql.org/) were chosen for the software implementation of the database.

For the design of the web resource, a client-server architecture was chosen, consisting of three main components: client, server and database. The Vue.js and Flask frameworks were used for development, and access management to the database is implemented through programmatic access based on REST technology.

Phylostratigraphic analysis and divergence analysis of genes associated with GMDs. Phylostratigraphic analysis is a method aimed at determining the evolutionary origin of genes by analyzing the presence of their orthologs, which are genes encoding homologous proteins that have diverged through speciation in the genomes of different species. This approach identifies key points in genome evolution, where a sharp increase occurred in the number of new genes, and helps to identify genes unique to specific taxa (Domazet- Lošo, Tautz, 2010).

We performed evolutionary analysis of genes included in the GlucoGenes® database using the phylostratigraphy age index (PAI) and divergency index (DI). The PAI value indicates how far the taxon reflecting the gene’s age is from the root of the phylogenetic tree (Mustafin et al., 2021). The taxon reflecting the gene’s age is considered as the taxon where the studied species diverged from the most distantly related taxon in which an ortholog of the gene has been found. The higher the PAI value of a gene, the younger it is. The Orthoweb software package (https://orthoweb. sysbio.cytogen.ru/run.html) was used for phylostratigraphic analysis. For PAI calculation, the method based on KEGG orthology groups was used (Kanehisa et al., 2016).

DI is an indicator of a gene’s evolutionary variability. DI is calculated based on the dN/dS ratio, where dN is the proportion of nonsynonymous substitutions in the DNA sequences of the studied gene and its ortholog; dS is the proportion of synonymous substitutions. This index was calculated by comparing human genes with genes from closely related organisms in the Hominidae family, specifically orthologs found in the western lowland gorilla Gorilla gorilla gorilla, Sumatran orangutan Pongo abelii, and common chimpanzee Pan troglodytes. The LPB93 model (Yang, 2007) was used to calculate dN/dS values. A DI value ranging from 0 to 1 indicates that a gene is undergoing stabilizing selection, a value of 1 indicates neutral evolution, and a value greater than 1 indicates positive selection.

Analysis of SNPs in 90-bp proximal regions of human gene promoters associated with GMD. In the Human_SNP_TATAdb knowledge base (Filonov et al., 2023), we searched for SNP variants in 90-bp proximal regions of human gene promoters associated with GMD that could statistically significantly decrease or increase the affinity of TATA-binding protein (TBP) to these promoters and consequently affect gene expression levels. Among all these SNPs, only those with clinical manifestations described in the publicly available ClinVar database (Landrum et al., 2014) were selected for further work. Finally, using the PubMed database, we performed curated annotations of how polymorphic changes in gene expression affected glucose levels in patients carrying these SNPs for all clinically relevant SNP markers located in promoters of genes associated with glucose metabolism disorders.

## Results

GlucoGenes® web resource

**Fig. 1.**
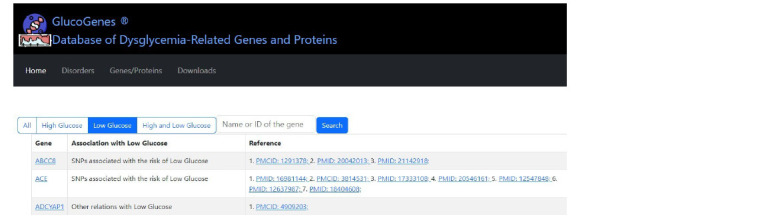
GlucoGenes® website interface.

The GlucoGenes® web resource is freely available at: https:// glucogenes.sysbio.ru/genes. The interface of the resource is shown in Fig. 1.

**Fig. 2.**
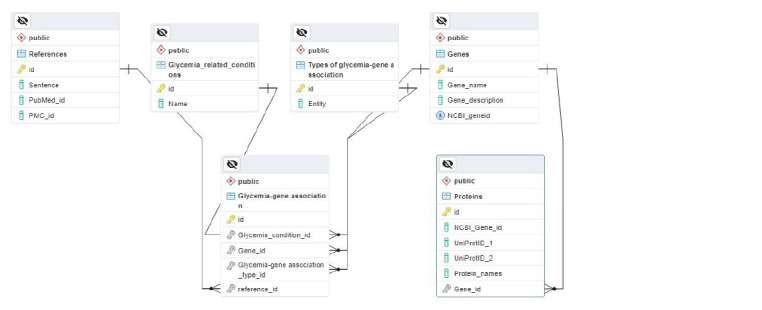
Entity-relationship diagram of the GlucoGenes® database.

The GlucoGenes® database consists of six components (tables). The Genes table contains gene names, descriptions and NCBI identifiers. The Proteins table includes protein names, descriptions, UniProt database identifiers, and links to the corresponding genes in the GlucoGenes® database. The Glycemia_related_conditions table provides information on glycemic disorders (hyperglycemia and hypoglycemia). The Types_of_glycemia_gene_association table contains information on the types of associations between molecules and glycemic disorders. The References table contains article identifiers in PubMed or PubMed Central with brief data extracts. The Glycemia_gene_association table is a summary table that aggregates information from all of the above-mentioned tables. The structure of the database is shown in Fig. 2. The database currently includes 561 genes associated with GMDs and 2,115 references to literature sources.

The GlucoGenes® web portal consists of four functional sections.

1. Homepage: The homepage provides general information about the resource and the terms used. We define hyperglycemia or high glucose levels in the culture medium as High Glucose (HG) and hypoglycemia or low glucose levels in the culture medium as Low Glucose (LG).

2. Disorders page: This page presents lists of genes associated with HG, LG, as well as with high and low glucose levels (HLG). For each gene, the type of association with glycemic disorders is indicated. The following categories of associations with glycemic disorders are highlighted: SNPs associated with HG, LG, or HLG; proteins with hyperglycemic activity; proteins with antihyperglycemic effects; genes up-regulated by HG; genes up-regulated by LG; genes down-regulated by HG; genes down-regulated by LG; and other associations with HG, LG, or HLG. For each gene and its association, references to relevant publications in PubMed are provided.

3. Genes/Proteins catalog: This section allows users to find gene names and NCBI gene identifiers, names of protein(s) encoded by the gene, and types of associations with GMD.

4. Downloads page: From this page, users can download lists of genes associated with HG, LG, and HLG, as well as all associated genes in Excel format. Search within the system is available by gene name, NCBI gene identifier, or type of GMD.

Data from the portal can also be accessed without using the graphical user interface via a REST application programming interface (API). This interface allows users to retrieve required information by sending a request to the web server in the form of a URL string. In response to such a request, the server returns results as a text page or file, where the information is structured according to the JavaScript Object Notation (JSON) format (http://json.org/). The resulting text file can be opened with any text editor. It can also be processed using various software tools, including user-written programs in general-purpose modeling environments (e. g., Matlab, Scilab) or high-level programming languages (e. g., Python, R, C++, Java).

An example of a REST request is given below (the result is a text file in structured JSON format): https://glucogenes. sysbio.ru/api/genes/<geneid> – returns a card with a description of the gene <geneid>.

**Fig. 3.**
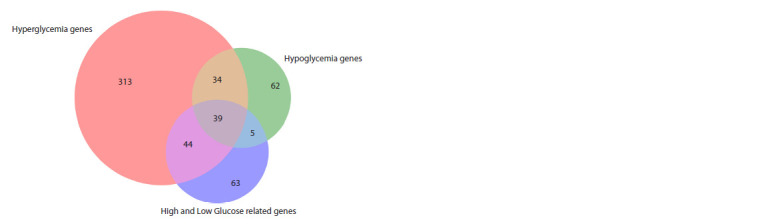
Venn diagram showing intersections of gene groups.

Evolutionary characteristics of genes associated with GMDs. We calculated PAI indices and plotted their distribution both for the list of protein-coding genes in the Homo sapiens genome and for genes represented in the GlucoGenes® database including those associated with hyperglycemia, hypoglycemia and glucose fluctuations (Table 1). Please, note that some genes were associated with more than one GMD (Fig. 3).

**Table 1.**
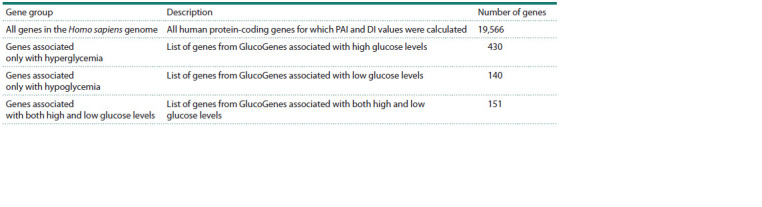
Lists of human protein-coding genes analyzed through phylostratigraphic analysis

**Fig. 4.**
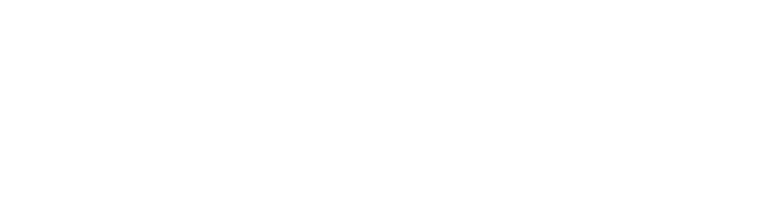
Distribution of protein-coding genes associated with GMDs by PAI values. Here and in Fig. 5: a – all human protein-coding genes (All_CDS) as a control group compared to genes associated with high glucose levels (Hyperglycemia); b – all human protein-coding genes (All_CDS) as a control group compared to genes associated with low glucose levels (Hypoglycemia); c – all human protein-coding genes (All_CDS) as a control group compared to genes associated with both high and low glucose levels (High and Low Glucose-related genes). Columns marked with asterisks indicate statistically significant differences between gene samples from the database and the sample of all human protein-coding genes: *p-value <0.05, ***p-value < 0.001. Statistical testing was performed using the chi-square test.

The distribution of PAI values for all genes in the human genome is multimodal, with two pronounced peaks at the levels of Cellular Organisms, Metazoa and Vertebrata- Euteleostomi (Fig. 4). The first peak is the largest; almost 55 % of genes in the H. sapiens genome have a PAI between 1 and 3. The second peak covers 32 % of the genes.

The results of the analysis showed a significant increase (up to 40 %) in the proportion of genes involved in glucose regulation with a PAI index = 3 in all three categories (Fig. 4). In particular, this group includes the TCF7L2, PPARG, GCGR, IRS1 and MTNR1B genes, the products of which are important regulators of glucose metabolism.

**Fig. 5.**
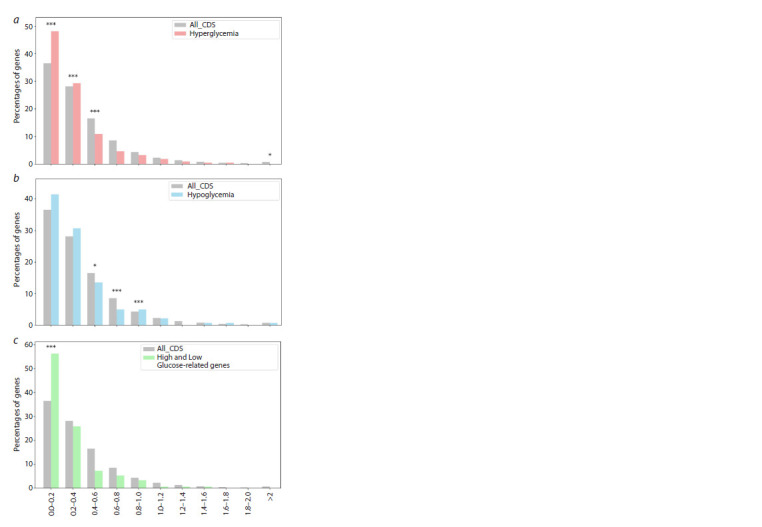
Distribution of protein-coding genes associated with GMD by DI values.

Sequence conservation analysis for the same gene lists (Fig. 5) showed that most of the genes studied are highly conserved (DI < 0.6) or conserved (DI < 1). This indicates the conservation of their functions during evolution and highlights their critical role in biological processes related to glucose regulation. However, several genes with a DI greater than 1 were identified, indicating recent exposure to positive selection. These genes include SPP1, CALCA, CD33, SULT2A1, TNF, ECM1, CYP3A4 and EDN1.

Analysis of SNPs in 90-bp proximal regions of human gene promoters associated with GMD. A total of 181 SNP markers were identified in the GlucoGenes ® database, which may either decrease (45 SNP markers) or increase (136 SNP markers) the expression of 52 human genes, thereby altering glucose levels in patients carrying minor alleles of these SNPs. Table 2 provides an example of 10 SNPs located in the promoters of the human ABCC8, INSR, and PGM1 genes, available in the ClinVar database (Landrum et al., 2014).

**Table 2.**
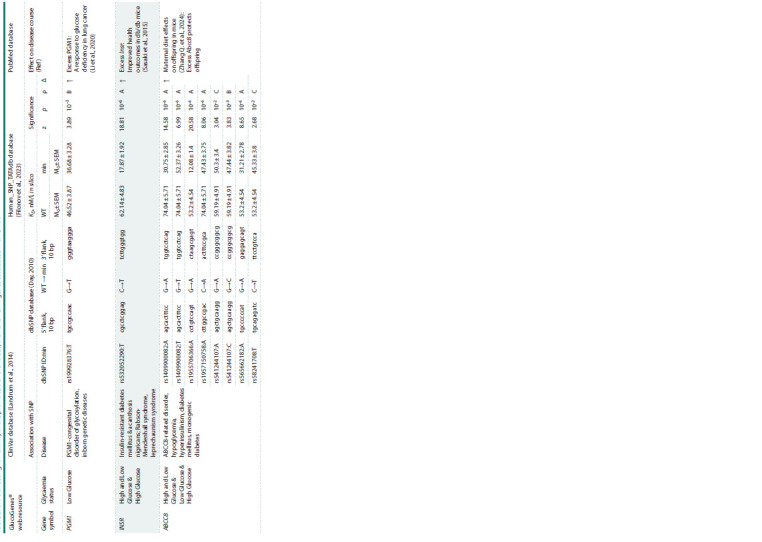
SNPs affecting TBP affinity in the promoters of the ABCC8, INSR and PGM1 genes associated with GMDs Notе. WT and min – ancestral (wild-type) and minor (pathological) alleles of SNPs; KD – equilibrium dissociation constant of the TBP-promoter complex, expressed in nanomoles per liter, nM/l; M0 and SEM – context-dependent in silico assessment and its standardized error; z, p and ρ – Fisher’s z-statistic, its significance level, and heuristic prioritization of in silico evaluations from best (A) to worst (D) in alphabetical order; Δ – expression increased (↑) or decreased (↓).

According to the data presented in Table 2, minor alleles of the ABCC8, INSR, and PGM1 gene promoters exhibit altered affinity for TBP, which may affect the expression levels of these genes and explain their association with GMDs. More detailed information on the identified SNP markers can be found in Supplementary Material1.


Supplementary Materials are available in the online version of the paper: https://vavilov.elpub.ru/jour/manager/files/Suppl_Klimontov_Engl_28_8.pdf


## Discussion

Advances in the study of the molecular biology of diabetes open up broad opportunities for the implementation of precision medicine technologies in the treatment of this disease. In particular, the identification of disease-specific biomarkers offers new prospects for diagnosis, monitoring, prognosis of the disease and its outcomes, pharmacogenetics of modern glucose-lowering drugs, as well as the search for new therapeutic agents (Chung et al., 2020). The rapid accumulation of data on the molecular basis of genetic predisposition to diabetes and the molecular mechanisms of its complications underscores the need for research tools to facilitate structured information retrieval in this field.

We have developed a database of genes and proteins that have demonstrated associations with GMDs, including hyperglycemia, hypoglycemia, or both. The web-based resource, named GlucoGenes® (https://glucogenes.sysbio. ru/genes/main), can be utilized to collect, search, and visualize information on genes and proteins associated with GMDs. Access to the database integrated into GlucoGenes® is provided via a REST-based API for record browsing. A graphical user interface allows users to view records and export their content in Excel format. The database contains catalogs of genes and proteins associated with GMDs, including information on the types of associations and links to abstracts of relevant publications in PubMed or full-text articles in PMC. Gene and protein lists are available for download. A limitation of this resource is that it accumulates data only from articles indexed in the PubMed and PMC. Regular information updates are evidently required.

The developed resource may prove useful for addressing research challenges in bioinformatics and the molecular biology of diabetes. Specifically, it can be applied to select genes and proteins for studying genetic predisposition to diabetes in various populations, investigating the molecular aspects of pathogenesis, searching for potential biomarkers of diabetic complications, identifying potential therapeutic targets, and other tasks. In this study, we present examples of using the developed resource to solve research tasks in bioinformatics studies.

The first task focuses on the evolutionary origin of genes associated with GMDs. Evolutionary analysis of genes using phylostratigraphy is a key tool in biology, enabling an understanding of the fundamental mechanisms underlying the diversity of life on Earth. The evolutionary history of genes provides insights into how various functions and structures have evolved and adapted to environmental changes. This knowledge not only aids in reconstructing phylogenetic trees but also helps to identify genes responsible for adaptive changes and specific physiological processes, such as glucose metabolism. The conducted phylostratigraphic analysis revealed that among genes associated with glucose metabolism, a significant proportion (up to 40 %) are genes with PAI = 3, corresponding to the origin of multicellular organisms (Maloof et al., 2010). Most of the studied genes were found to be highly conserved (DI < 0.6) or conserved (DI < 1). The obtained results emphasize the importance of GMD-associated genes in regulating specialized metabolic processes characteristic of complex organisms.

During the second task, data from the web resource were used to analyze SNPs in proximal regions of human gene promoters that affect the affinity for TBP. The integration of GlucoGenes® data with information on SNP associations with various human diseases from other databases, on the one hand, and bioinformatic assessments of changes in glucose levels in patients carrying these SNPs, on the other hand, reflects the molecular mechanisms through which GMDs may influence the progression of these diseases.

## Conclusion

GlucoGenes® is a resource that combines a graphical user interface with a database of genes and proteins associated with hyperglycemia, hypoglycemia, and both metabolic disorders. The resource has been utilized for bioinformatic analysis of the evolutionary characteristics of genes associated with these disorders, as well as for the analysis of SNPs in proximal promoter regions of genes that affect the affinity for TBP. It has been demonstrated that a significant proportion of genes associated with GMDs are evolutionarily ancient and conserved. SNP markers that can decrease (45 SNP markers) or increase (136 SNP markers) the expression of 52 genes have been identified.

## Conflict of interest

The authors declare no conflict of interest.
